# A First Generation Comparative Chromosome Map between Guinea Pig (*Cavia porcellus*) and Humans

**DOI:** 10.1371/journal.pone.0127937

**Published:** 2015-05-26

**Authors:** Svetlana A. Romanenko, Polina L. Perelman, Vladimir A. Trifonov, Natalia A. Serdyukova, Tangliang Li, Beiyuan Fu, Patricia C. M. O’Brien, Bee L. Ng, Wenhui Nie, Thomas Liehr, Roscoe Stanyon, Alexander S. Graphodatsky, Fengtang Yang

**Affiliations:** 1 Institute of Molecular and Cellular Biology, SB RAS, Novosibirsk, Russia; 2 Novosibirsk State University, Novosibirsk, Russia; 3 State Key Laboratory of Genetic Resources and Evolution, Kunming Institute of Zoology, Chinese Academy of Sciences, Kunming, PR China; 4 Wellcome Trust Sanger Institute, Wellcome Trust Genome Campus, Hinxton, Cambridge CB10 1SA, United Kingdom; 5 Centre for Veterinary Science, Department of Veterinary Medicine, University of Cambridge, Cambridge, United Kingdom; 6 Jena University Hospital, Institute of Human Genetics and Anthropology, Jena, Germany; 7 Department of Biology, University of Florence, Florence, Italy; Texas A&M University, UNITED STATES

## Abstract

The domesticated guinea pig, *Cavia porcellus* (Hystricomorpha, Rodentia), is an important laboratory species and a model for a number of human diseases. Nevertheless, genomic tools for this species are lacking; even its karyotype is poorly characterized. The guinea pig belongs to Hystricomorpha, a widespread and important group of rodents; so far the chromosomes of guinea pigs have not been compared with that of other hystricomorph species or with any other mammals. We generated full sets of chromosome-specific painting probes for the guinea pig by flow sorting and microdissection, and for the first time, mapped the chromosomal homologies between guinea pig and human by reciprocal chromosome painting. Our data demonstrate that the guinea pig karyotype has undergone extensive rearrangements: 78 synteny-conserved human autosomal segments were delimited in the guinea pig genome. The high rate of genome evolution in the guinea pig may explain why the HSA7/16 and HSA16/19 associations presumed ancestral for eutherians and the three syntenic associations (HSA1/10, 3/19, and 9/11) considered ancestral for rodents were not found in *C*. *porcellus*. The comparative chromosome map presented here is a starting point for further development of physical and genetic maps of the guinea pig as well as an aid for genome assembly assignment to specific chromosomes. Furthermore, the comparative mapping will allow a transfer of gene map data from other species. The probes developed here provide a genomic toolkit, which will make the guinea pig a key species to unravel the evolutionary biology of the Hystricomorph rodents.

## Introduction

The domesticated guinea pig (*Cavia porcellus*) is a proverbial animal model traditionally used in biomedical research (e.g. [1,2]). The correct diploid number (2n = 64) of *C*. *porcellus* was determined more than 60 years ago [3]. Before banding or differential staining became available variants of several chromosomal pairs of guinea pig were reported in the literature [4,5,6,7]. With the advent of C-banding it became clear that these variants were due to differences in the amount of heterochromatin [8]. Later there were a series of more detailed reports on differential staining chromosomes of the chromosomes of *C*. *porcellus* [9,10,11,12,13,14,15,16]. However, in spite of the guinea pig's importance in research there is no karyotype standard or chromosome nomenclature for this species.

The lack of genomic resources for the guinea pig compared to other rodents such as the laboratory mouse and rat explains why, over the last decade, the guinea pig has fallen out of favor as a model organism. In contrast, the laboratory rat and mouse were among the first organisms for which high coverage genome assemblies were available.

A low coverage (7x) of the guinea pig genome assembly only became available in 2008 as one of the 29 mammals whose genomes were sequenced by the Mammalian Genome Project [17,18]. Additional sequencing of several guinea pig strains is under way for SNP discovery [18,19]. However, the guinea pig sequence scaffolds have not been anchored to chromosomes in the current assembly, largely due to the lack of large-insert clone-based physical maps (although BACs are available for the guinea pig [20]) and maps of conserved synteny. A well-characterized karyotype and map of conserved synteny with human is the first step towards linking sequencing data and chromosomes.

Comparative cytogenetic maps enable homology links between genomes and transfer of gene mapping information from the well-studied genomes to uncharted genomes of other organisms [21]. Comprehensive chromosome painting data is available to comparatively link the human and representatives of all major mammalian clades (cf. [22,23]). Although comparisons between human and rodent genomes using chromosome painting have had limited success, results in Sciuromorpha, Castorimorpha, and Anomaluromorpha showed that most of the ancestral eutherian syntenic associations were conserved [24,25,26,27,28,29,30,31]. However, some rodents such as Myomorpha have experienced massive chromosomal rearrangements [32]. Currently there is no information about the rate of genomic changes in Hystricomorpha, the taxa to which *C*. *porcellus*, belongs. It is thus pertinent and timely to extend chromosome painting to a Hystricomorpha rodent and in particular the guinea pig.

Chromosome painting maps are most often unidirectional. However, cross-species reciprocal painting is more precise and can generate detailed subchromosomal-level comparative maps. In reciprocal painting probes are produced from both species and painting is bi-directional. Currently, painting probe sets are available for only 19 rodent species and, there are only a few reports of reciprocal painting studies in rodents [33,34,35,36,37,38,39]. In spite of the fact that the Hystricomorpha comprises over 260 species up to now only two paint sets were produced (*Octodon degus* [40] and *Heterocephalus glaber* [41]). Further, there is still no consensus for the phylogenetic relationship between the guinea pig and other hystricomorphs [42,43,44]. The generation of a set of paint probes for the guinea pig will help resolve phylogenetic relationships within Hystricomorpha, especially when integrated with other biomolecular results [42,44,45,46].

Here, painting probes for domesticated guinea pig were made from two fibroblast cell lines by flow sorting and microdissection. The application of the probes to cross-species reciprocal chromosome painting with human allowed us to establish a comparative chromosome map between domesticated guinea pig and human.

## Materials and Methods

### Ethics statement

Two primary fibroblast cell lines of *C*. *porcellus* (male) were used in this study: the first is an established cell line (Cat number: KBC 200301) that was obtained directly from Kunming Cell Bank (KCB), the Chinese Academy of Sciences, while the second was derived from skin biopsy from a guinea pig obtained from the animal facility at the National Cancer Institute (NCI), USA. For convenience, the former cell line was named as CPO-KCB, while the latter as CPO-NCI.

Tissue sample for CPO-NCI was obtained in strict accordance with the recommendations in the Guide for the Care and Use of Laboratory Animals of the National Institutes of Health. The animals were not sacrificed. Instead a small piece of ear, about 1–2 mm square was cut with scissors from the tip of the ear as described in [47]. It was not necessary to use anesthesia or analgesics as very minimal damage or stress was done to the animals and they were immediately released with no ill effects.

### Chromosome preparation and chromosome staining

Human metaphases were prepared from a short-term culture of human peripheral lymphocytes stimulated with a combination of three mitogens: pokeweed (Sigma-Aldrich, final concentration: 1%), phytohemagglutinin (Sigma-Aldrich, final concentration: 1%) and conconavalin A (Sigma-Aldrich, final concentration: 1%). The cultures were arrested with colchicine (final concentration: 0.15 μg/ml) for 45 min and chromosomes were harvested using a standard procedure [9].

The *C*. *porcellus* cells were cultivated and chromosomal suspensions were made as described previously [47,48]. Metaphase preparations were made as described earlier [49,50]. The GTG- and CBG-bandings were performed as described by [51] and [52], respectively.

### Generation of painting probes for *C*. *porcellus*

The set of human chromosome specific painting probes has been generated in the Cambridge Resource Centre for Comparative Genomics (UK) and provided for collaborative research use [53].

Painting probes for *C*. *porcellus* were generated independently from the two cell lines mainly by flow sorting. The flow sorting of CPO-KCB was done using a FACStar Plus (Becton Dickinson) at the University of Cambridge [54] as well as using a MoFlo Cell Sorter (Beckman Coulter) at the Wellcome Trust Sanger Institute (UK) [55]. Painting probes from both CPO-KCB were made using the conventional 6-MW primer (5’-CCG ACT CGA GNN NNN NAT GTG G-3’) and were labeled with biotin- and digoxigenin-dUTP (Roche) as well or directly with Cy3-, Cy5- or Green-dUTPs (Jena BioScience) by DOP-PCR [54,56]. The chromosomes of CPO-NCI were sorted using FACS Vantage SE (Becton Dickinson) at the National Cancer Institute (USA) [57]. Four different DOP-primers (6MW, FS (5′-CGG ACT CGA GNN NNN NTA CAC C-3′), GAG (5′-GAG GAG GAG GAG GAG GAG GAG -3′), G1/G2 (5′-GAG GAT GAG GTT GAG NNN NNN TGG-3′/5′-GTG AGT GAG AGG ATG AGG TTG AG-3′)) were used for chromosome test sorts. As G1/G2 primers produced the best paints with a minimum of cross hybridization they were selected to amplify the entire set of flow-sorted chromosomes. First round of PCR was made with primer G1 and the second round of PCR with primer G2 [57].

Microdissection was used to generate chromosome-specific probes for such CPO-NCI chromosomes that could not be resolved into single-chromosome-containing peaks by flow cytometry at the Institute of Human Genetics and Anthropology (Germany) as previously described [58]. All microdissected probes except CPO Xp and Xq were generated using GenomePlex Whole Genome Amplification (WGA1) Kit (Sigma-Aldrich) [59]. For CPO Xp and Xq generation DOP-PCR was used.

### Telomeric and ribosomal DNA probes

The telomeric DNA probe was generated by PCR using the oligonucleotides (TTAGGG)_5_ and (CCCTAA)_5_ [60]. Clones of human ribosomal DNA containing the complete 18S-rRNA and 28S-rRNA genes were obtained as described [61].

### Fluorescence *in situ* hybridization (FISH)

We used sequential GTG-banding [51] and FISH or simultaneous DAPI-banding and FISH for precise chromosome identification [49,50]. Four digital imaging systems were used in this study: VideoTesT-FISH and VideoTesT-Karyo (VideoTesT, Saint-Petersburg, Russia), Case Data Manager (Applied Spectral Imaging Inc.), CytoVision system (Applied Imaging Corp.), and SmartCapture and SmartType Karyotyper (Digital Scientific, UK). Hybridization signals were assigned to specific chromosome regions defined by GTG- or DAPI-banding patterns.

## Results

### Generation of chromosome-specific painting probes and assignment of probes onto G-banded chromosomes

**Fig 1:**
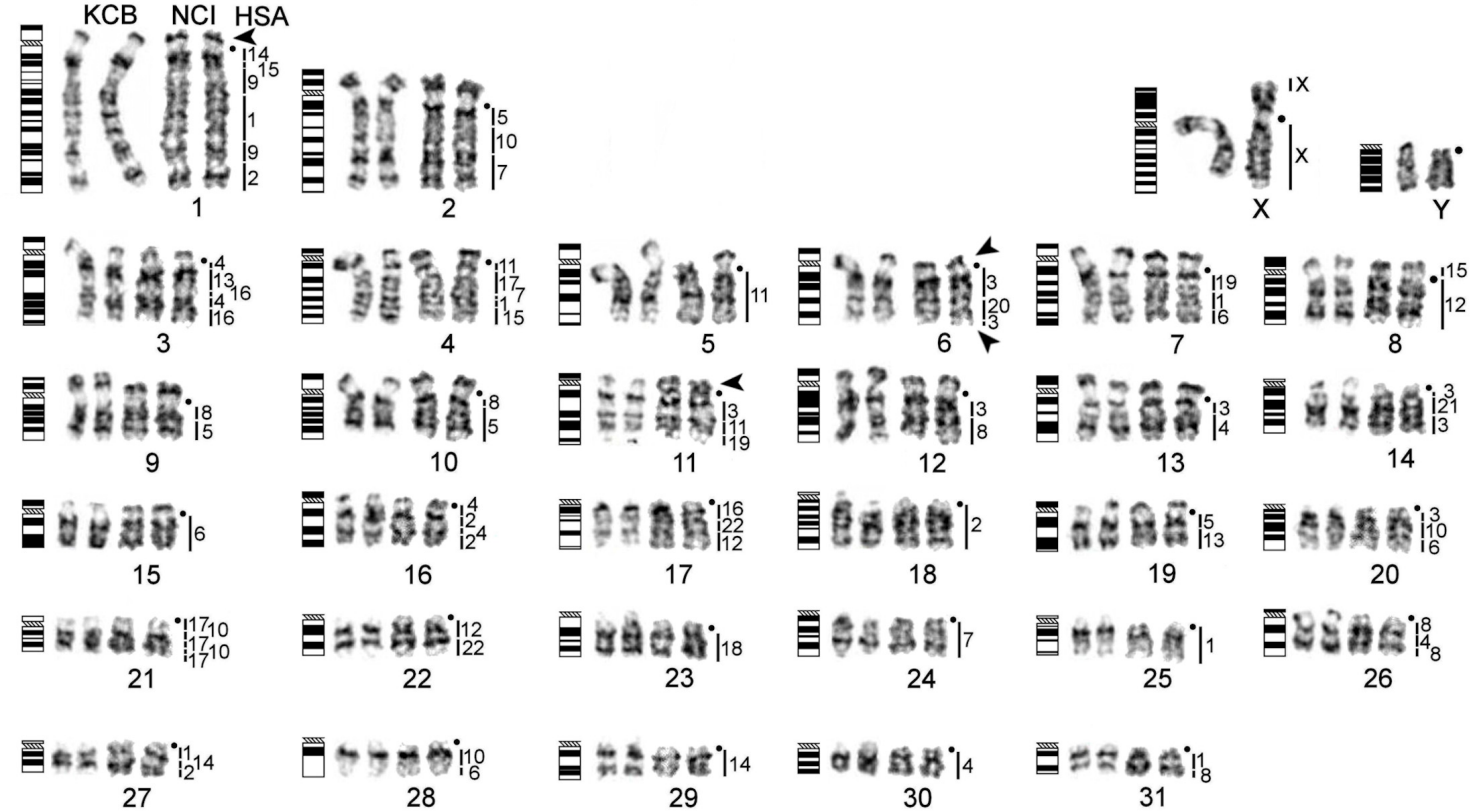
Idiogram and karyotype of the guinea pig with homologies to human (HSA) revealed by comparative chromosome painting. Black dots mark position of centromeres. Black triangles indicate NORs located on CPO1, CPO6 and CPO11 (see comments in the text). Each pair presented in two copies to show chromosomes with different resolution: KCB—CPO-KCB, NCI—KPO-NCI.

**Fig 2:**
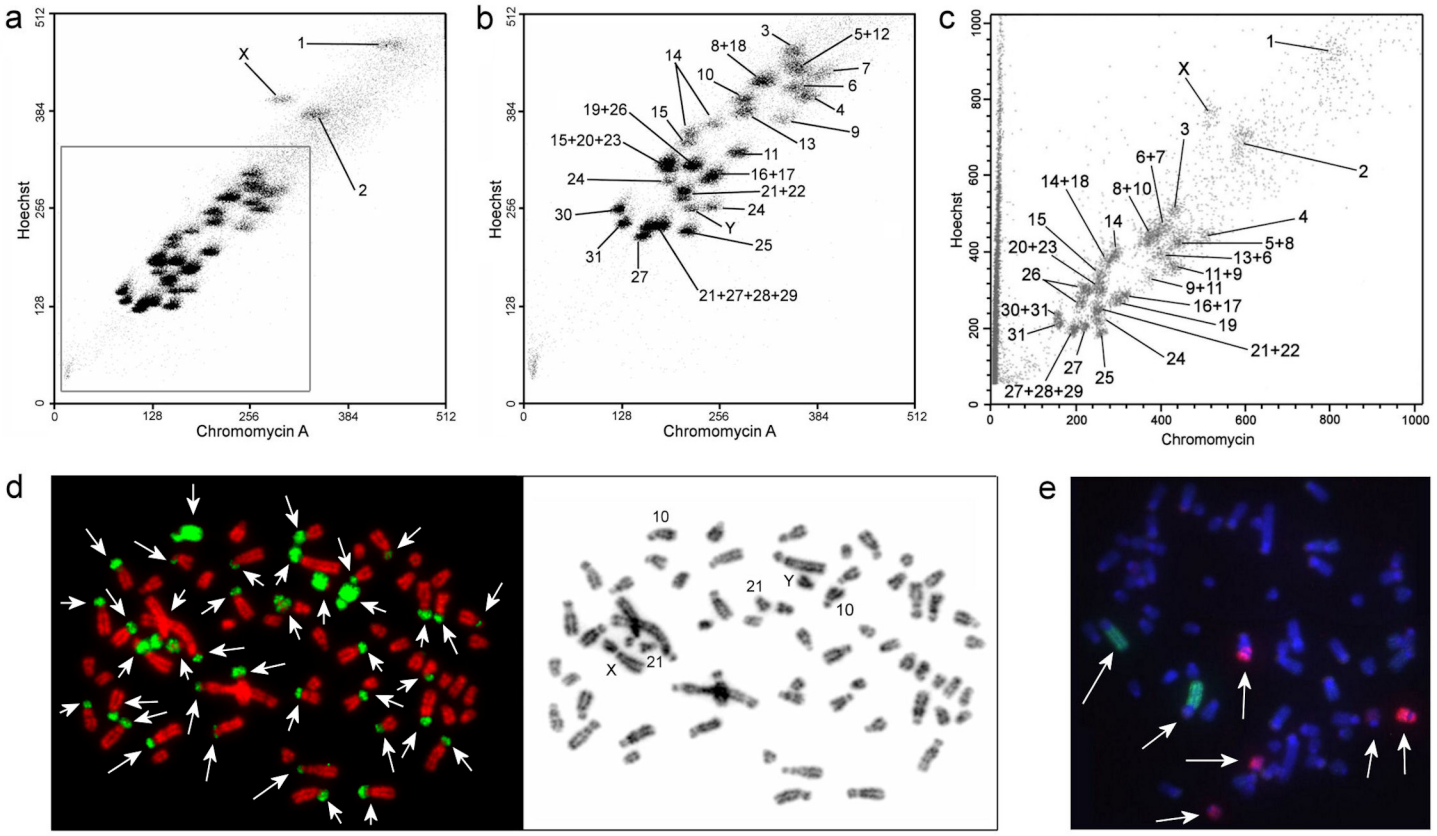
Bivariate flow karyotypes of *C*. *porcellus* and characteristic hybridization patterns of paint probes. a and b—Flow karyotype of CPO-KCB cell line, a shows the annotated top chromosomes and b show an enlarged version of the inferior part of the flow karyotype (in grey frame), c—CPO-NCI cell line, d—hybridization pattern of CPO10 paint generated using 6MW primer, e— CPO2 (green) and CPO14+18 (red) paints (indicated by arrows) from CPO-NCI cell line generated with G1 and G2 primers on metaphase chromosomes from *C*. *porcellus*. Arrows indicate the painting results in Fig 2d and 2e.

The karyotype of *C*. *porcellus* contained many chromosomes of similar sizes and morphology (Fig 1), which represented a technical challenge for chromosome sorting by flow cytometry. Chromosome suspensions from the two cell lines of *C*. *porcellus* (CPO-KCB and CPO-NCI) were independently subjected to flow-sorting (Fig 2A–2C), which returned two sets of probes, each with its own characteristics. The chromosomes of CPO-KCB were resolved into 28 peaks (Fig 2A and 2B). The set of probes from CPO-KCB generated by DOP-PCR with the 6MW primer often gave strong cross-hybridization signals onto the short arms of the biarmed autosomes as well as to the largely C-band positive regions on chromosomes 21, Xp-proximal, and Yq, as demonstrated by the hybridization patters of CPO10 (Fig 2D). These probes were identified mainly by sequential G-banding and multi-colour FISH. The quality of this set of probes varied when applied to cross-species painting; for probes that did not work well when painted onto human chromosomes, new probes were made using a new batch of flow-sorted chromosomes. Although CPO-KCB was sorted using two different cell sorters (i.e. FACStar Plus vs. MoFlo), no obvious difference in resolution was found in the flow karyotypes.

Two separate attempts were made to sort the chromosomes of CPO-NCI, resolving the chromosomes into 26 peaks (Fig 2C) and 28 peaks, respectively. Applying a tighter sorting gate on the flow chart only led to a slight reduction in the number of peaks containing multi-chromosomes. The content of each peak was determined by hybridizing the paint probes back onto G-banded chromosomes of the *C*. *porcellus*. Both sets of probes made using the G1 and G2 primer did not produce cross hybridization signals to the C-band positive heterochromatic blocks and the repeat-rich short arms of the biarmed autosomes (Fig 2E). Indeed, at NCI, Frederick, USA, chromosome test sorts were first amplified by a number of different DOP-primers (6MW, FS, GAG, G1/G2). Then the primer that produced the best paints with a minimum of cross hybridization was selected to amplify the entire set of flow-sorted chromosomes. In this case the cleanest results were produced by DOP-PCR with G1 and G2 primers which enabled the avoidance of excessive amplification of heterochromatin. However, chromosomes 12 and Y were apparently “missing” from the flow karyotype of CPO-NCI, perhaps due the use of over-tight sorting gates.

The GTG-banded karyotype *of C*. *porcellus* has been reported before [9,10,12,14,15]. Nevertheless, apart from a few large-sized chromosomes and X, the majority of the medium- and small-sized chromosomes were almost impossible to identify unambiguously by banding patterns alone. Furthermore, as demonstrated by flow cytometry (Fig 2A–2C), the same chromosomes, due to the variation in heterochromatin, often appeared in different positions in the two flow karyotypes. Notably, the positions of most medium-sized chromosomes (CPO 4–24) in the two flow karyotypes (Fig 2A–2C) often appeared to be “inconsistent” when they were compared across the two cell lines. Nevertheless, such an apparent inconsistency was due to variations in the heterochromatic short arms among individuals and as such was true reflection of chromosome short arm heteromorphisms between individuals [8]. It was difficult to estabish the correspondence between all the chromosomes of CPO-KCB and CPO-NCI on the basis of G-banding and painting (Fig 1). Indeed, it was possible to unambiguously establish the G-banded chromosome correspondence between some chromosomes only after hybridizing both sets of painting probes onto human metaphases. Here we opted to establish a new chromosome nomenclature, because it was impossible to follow published karyotypes with confidence. We arranged chromosomes mainly according to size.

Since the majority of the *C*. *porcellus* chromosomes could not be unequivocally identified by GTG- or DAPI-banding alone and many autosomes could not be sorted separately, we also made a set of probes derived from microdissected CPO-NCI chromosomes, in order to increase the resolution of *C*. *porcellus* probes. We generated six subchromosome specific probes for the three largest CPO chromosomes—CPO1, 2 and X: for chromosomes CPO1 and CPO2 we obtained probes for proximal and distal parts of the q-arms; while for the X chromosome we prepared probes for p- and q-arms. The p-arm of the X chromosome contains a huge heterochromatic block, giving a strong hybridization signal on CPOY heterochromatin. Chromosome-specific probes were also made by microdissection of 16 autosomes (CPO5, 6, 7, 9, 10, 11, 13, 14, 17, 20, 21, 22, 23, 27, 29 and 30). The quality of probes varied, with some highlighting the heterochromatic regions on other chromosomes, but they were useful for reliable identification of co-sorted CPO chromosomes.

### Standardization of *C. porcellus* karyotype

The precise comparison of GTG-banded chromosomes obtained in different laboratories was the first step in the construction of the comparative chromosome map for human and guinea pig. Summarizing the guinea pig karyotype description based on published data and presented here we can conclude that: the diploid chromosome number is 2n = 64 and the fundamental number of chromosomal arms is FN = 92. The X is a medium size submetacentric and the Y is a small size acrocentric with a large block of heterochromatin on the q-arm. Some chromosome pairs are NOR-bearing (see below) and several chromosomal pairs possess large blocks of heterochromatin with a variation in size even between homologues. Telomeric repeats are concentrated in pericentromeric regions. To solve the problem of chromosome correspondence in different reports in the literature we introduce here a nomenclature of *C*. *porcellus* GTG-banded chromosomes. The nomenclature is made in reference to an ideogram of *C*. *porcellus* chromosomes and in reference to human chromosome homology (Fig 1, S1 Fig). All pairs of autosomes were placed in order of decreasing size. Additionally, development of a set of molecular markers (for example, BAC markers) for easy molecular identification of each chromosome through FISH would be beneficial for *Cavia* cytogenetics. For now the combined use of *Cavia* chromosome-specific probes developed here and human chromosome probes has enabled the unambiguous identification of each of the guinea pig chromosomes.

### CBG-banding in CPO-NCI

**Fig 3:**
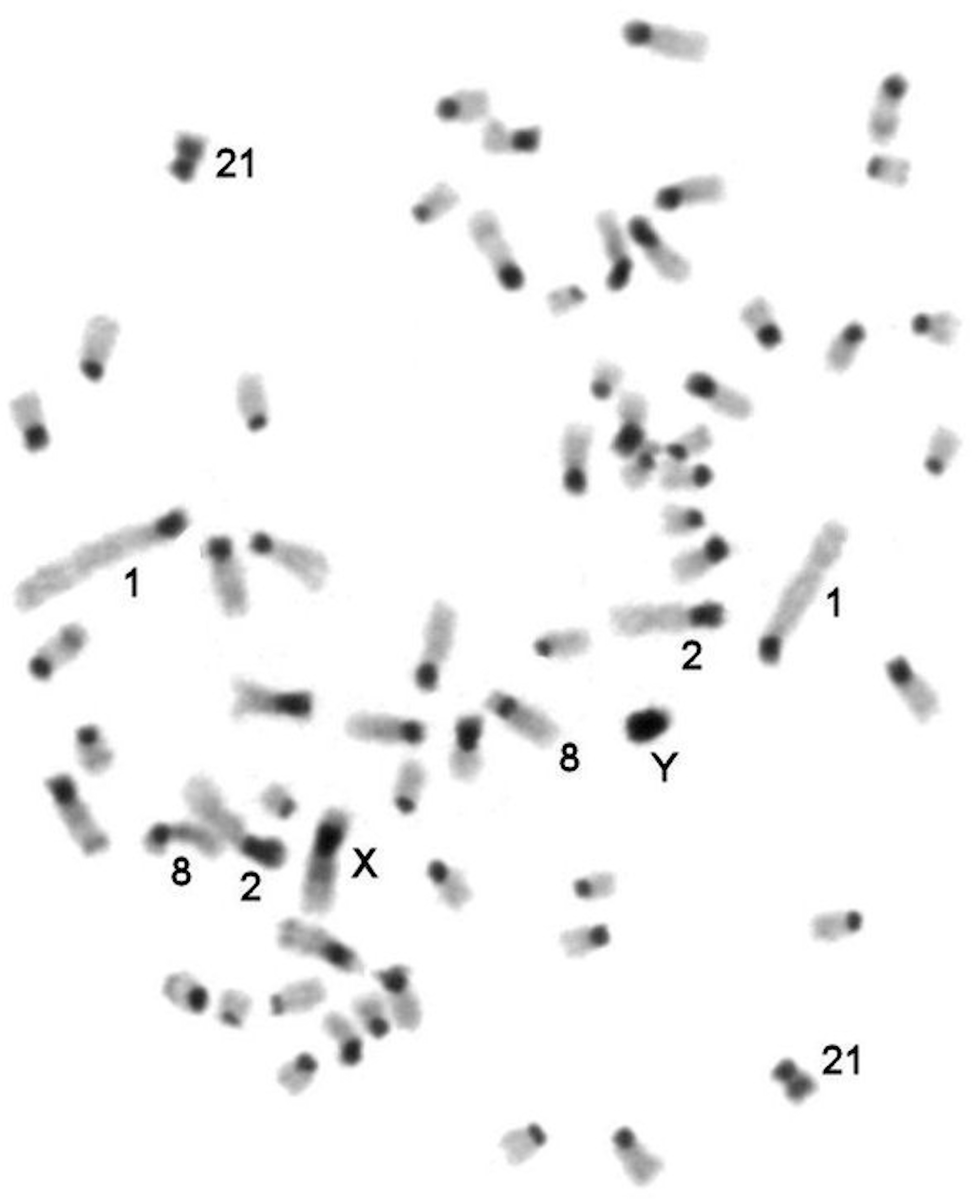
CBG-banding of *C*. *porcellus* metaphase chromosomes. Figures were constructed according to chromosome size and CBG-banding.

We observed the same CBG-banding pattern for *C*. *porcellus* chromosomes, as that reported previously [9,13,14]. CBG-banding revealed that all autosomes in *C*. *porcellus* karyotype had rather large C-positive pericentromeric blocks. A pair of small-sized autosomes (CPO21) had large blocks of pericentromeric heterochromatin on both arms. The *C*. *porcellus* Y-chromosome was C-positive and X-chromosomes had pericentromeric blocks on the p-arms (Fig 3).

### Distribution pattern of telomeric repeats and ribosomal DNA in CPO-NCI

**Fig 4:**
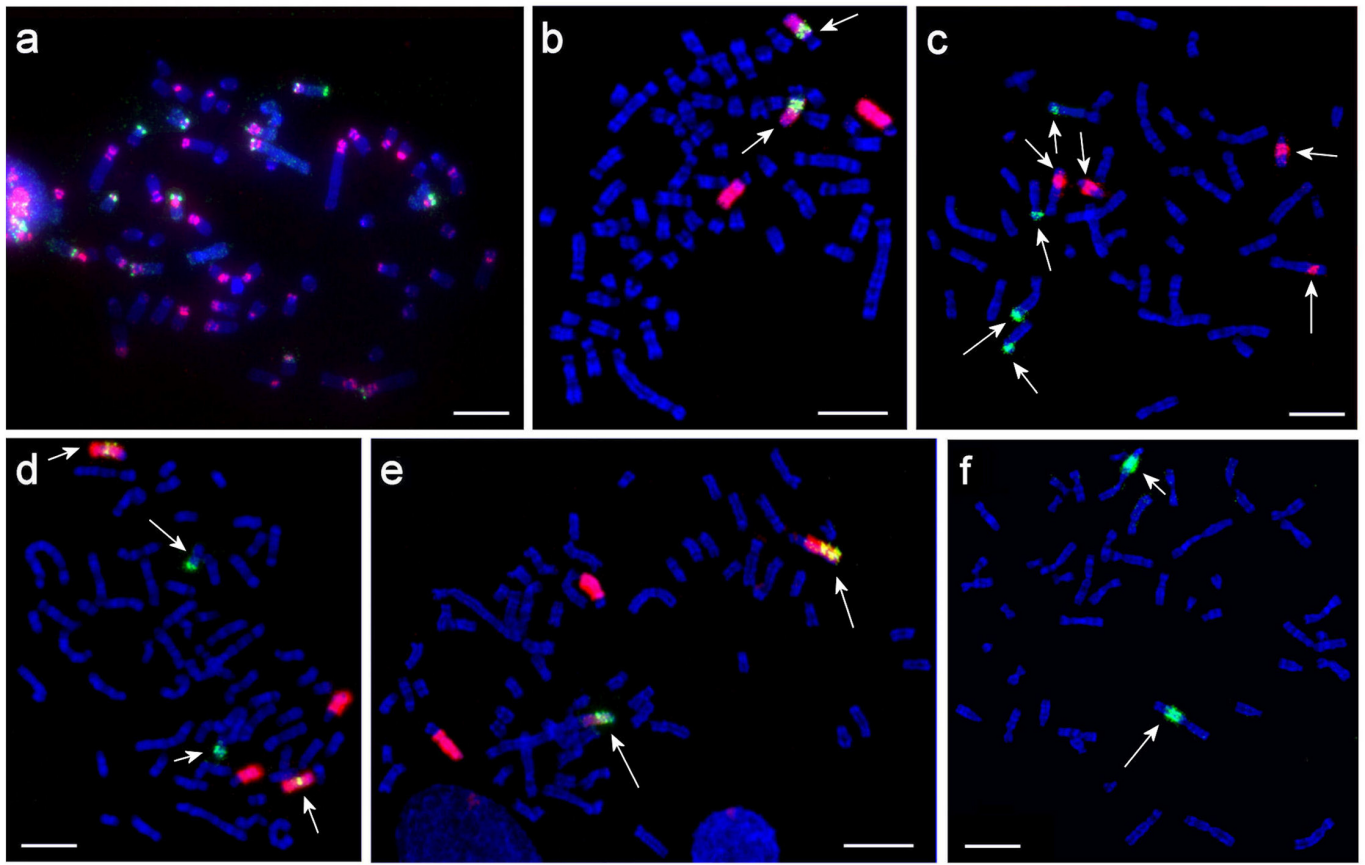
Examples of fluorescent *in situ* hybridization. a—localization of telomeric DNA probe (red) and rDNA probe (green) on metaphase chromosomes from *C*. *porcellus*; telomeric signals are too weak to capture without risking over-exposure to the strong signals from pericentromeric regions; b—HSA19 (green) and CPO6+7 (red) on metaphase chromosome from *C*. *porcellus*; c—CPO26 (green) and CPO19 (red) on metaphase chromosome from *H*. *sapiens*; d—HSA22 (green) and CPO14+16 (red) on metaphase chromosomes from *C*. *porcellus*; e—HSA20 (green) and CPO6+7 (red) on metaphase chromosomes chromosome from *C*. *porcellus*; f—CPO14 on metaphase chromosomes from *H*. *sapiens*. Arrows indicate the corresponding human chromosomes in 4b, 4d, 4e and the corresponding *C*. *porcellus* chromosomes in 4c and 4f. Scale bars indicate 10 μm.

Clusters of telemetric sequences were found not only at telomeres of chromosomes but were also present at the large pericentromeric regions and heterochromatic blocks in addition to the main pattern of localization of telomeric repeats as reported by Meyne *et al*. ([62]). The use of early metaphases with longer chromosomes allowed the detection of some more detailed features, revealing one to three clusters on each chromosome at the pericentromeric region. We localized the 18S-rRNA/28S-rRNA probe by itself and in dual-color FISH with a telomeric probe (Fig 4A). We observed unusual intermittent patterns of telomeric and NOR signals on several chromosomes. For example, a NOR co-localized with a large telomeric block on the distal part of the p-arm on CPO1. But it apparently shows some heteromorphism between two homologues of one pair: the NOR signal is distinctly seen on one homologue and is covered by the telomeric signal on the other. Possibly it explains the heteromorphism of CPO1 p-arm described in earlier works on the *C*. *porcellus* karyotype [7,63]. Our NOR localization through FISH following GTG-banding and co-localization with selected chromosome-specific probes allowed us to determine that chromosomes 1 and 11 carry NOR on the short arm. While most of the NORs are located on the distal part of p-arms, one chromosome (CPO6) appears to have a NOR on both p- and q-arms (Fig 4A). In all we observed 7–8 pares of NOR-bearing chromosomes. All but one NOR-bearing pairs of chromosomes carry large clusters of telomeric sequences. Additional studies are required to determine the precise localization of other NOR sites.

### Reciprocal chromosome painting between human and *C. porcellus*

**Fig 5:**
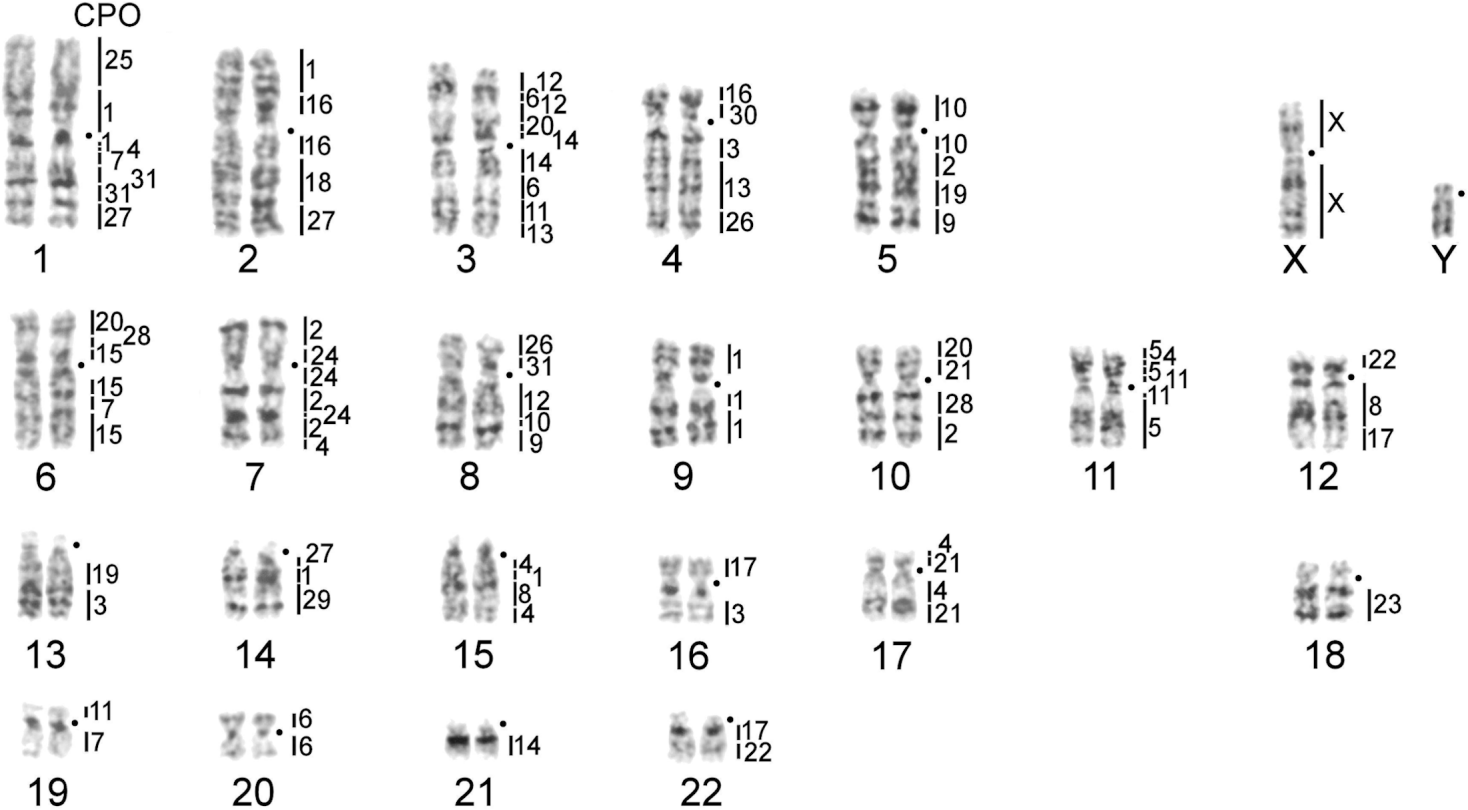
Human karyotype with homologies to guinea pig (CPO) revealed by painting. Black dots mark positions of centromeres.

Reciprocal painting between human and *C*. *porcellus* generated high-resolution comparative chromosome maps. The hybridization of 22 human autosomal probes onto the *C*. *porcellus* metaphase chromosomes revealed 78 homologous segments in the *C*. *porcellus* genome (Fig 1). The following associations of synteny-conserved human chromosome segments were detected in the guinea pig genome: HSA 1/8, 1/14/2, 2/4/2/4, 2/9/1/9/15/14, 3/4, 3/8, 3/10/6, 3/11/19, 3/20/3, 3/21/3, 4/13/16/4/16, 5/8 (twice), 5/10/7, 5/13, 6/1/19, 6/10, 8/4/8, 11/17/7/1/15, 12/15, 12/22, 12/22/16, 17/10/17/10/17 (Fig 1). The “reverse” painting from *C*. *porcellus* autosomal probes (see examples of fluorescence *in situ* hybridization in Fig 4B–4F) also detected 78 conserved synteny segments in the human genome (Fig 5). Paints made from both *C*. *porcellus* lines were hybridized onto human chromosomes, which also served as a common reference point for double checking the correspondence of flow peaks in the flow karyotypes of CPO-NCI and CPO-KCB. As expected, the heterochromatic regions on both human and *C*. *porcellus* chromosomes were not hybridized by any chromosomal probes in reciprocal painting between distantly related species (or cross-order reciprocal painting).

## Discussion

The guinea pig, *Cavia porcellus*, was one of the most important biomedical animal models in the last century. It lost popularity in part due to a lack of modern genomic tools to fully exploit this animal model. In contrast, the mouse was the first mammalian species after human (2001) for which, only one year later (2002), a complete genome assembly was available. The genome of guinea pig was only sequenced in 2008, but without even assigning scaffolds to actual chromosomes. There was no data to relate the karyotype of the guinea pig to human before this report.

Here we generated sets of chromosome painting probes for the guinea pig (*C*. *porcellus)* and established homology maps between this species, human and by extension other rodents. The great phylogenetic distance between hystricomorph rodents and primates (the divergence time between the ancestor of rodents and the common ancestor of primates and artiodactyls is about 80–100 Myr [64,65]), together with extensive rearrangements between humans and *C*. *porcellus*, made the FISH-comparison difficult. Due to their similar size multiple chromosomes were found in many peaks of the flow karyotype. Chromosomes were difficult to cytogenetically identify due to similar size and shape, a lack of distinctive banding patterns, and in some case, hybridization quality. Only the combination of expertise and resources of different laboratories enabled us to overcome these difficulties and achieve a full and precise comparison between the karyotypes of guinea pig and human.

### Ancestral associations found in the guinea pig

Among associations of synteny-conserved human chromosome segments detected in the guinea pig genome by reciprocal chromosome painting (Fig 1) only five of them are common for the guinea pig and the putative eutherian/rodent ancestral karyotypes [25]: HSA 3/21, 8/4/8, 12/22 (twice), and 14/15. It is very likely that these syntenies are homologous in the guinea pig and other rodents; however, only future research will verify if these breakpoints and syntenies have identical origins. In contrast, the HSA7/16 and 16/19 associations presumed ancestral for eutherians were not found. Furthermore, three syntenic associations (HSA1/10, 3/19, and 9/11) considered ancestral for rodents were also not found in *C*. *porcellus*. The most likely hypotheses to explain these findings are that 1) the associations were lost in the evolutionary line leading to the guinea pig due to a high rate of chromosome evolution, or 2) the size of the associations in the guinea pig were below the resolution of techniques used in our study.

### Level of karyotype divergence in the guinea pig

The number of autosomal conserved segments between two species can serve as a measure of karyotype divergence. Rodents branched out of the mammalian tree about 100 MYA according to molecular clock estimates, and after another 40 MYA Hystricomorpha have diverged from other rodent suborders [66]. In the order Rodentia the number of conservative segments revealed by human painting probes varies from 36–37 fragments in squirrels to 95 in mouse and rat genomes [32]. This range of differences results from at least two modes of genome reorganization in rodents: a slow, conservative evolution in Sciuromorpha and a high evolutionary rate in Myomorpha which disrupted chromosomal syntenies.

Our reciprocal chromosome painting and GTG-banding comparisons between guinea pig and human allowed us to estimate the level of chromosomal divergence of the guinea pig karyotype. Using human painting probe we found a total of 78 segments of homology between guinea pig and human. We can conclude that the guinea pig has a high rate of chromosome evolution. This rate is comparable to but somewhat lower than that found in myomorphs (78 *vs* 95 segments found in mouse).

### Future use of guinea pig painting probes in phylogenomics

The painting probe sets developed from the guinea pig will also allow more incisive studies of hystricomorph chromosome evolution and allow comparison between hystricomorphs and other rodent taxa. There are very few studies that describe karyotypes of the closest relatives of guinea pig [67,68,69]. In general all members of the genus *Cavia* have a diploid number 2n = 64, except the island species *C*. *intermedia* with 2n = 62 [67]. Apparently there is a great deal of variation in the amount and localization of heterochromatic blocks between different species. To our knowledge, there are no studies that directly compared differentially stained chromosomes between Caviidae species. There are no studies comparing the guinea pig chromosomes with those of other mammals. Compared with all other rodent groups, Hystricomorpha, which include among others the porcupines, chinchillas, pacas, agoutis and capybaras, is far less well-studied by modern molecular cytogenetic approaches [40,41,67,70].

We hypothesize that hystricomorph rodents in general are probably characterized by much higher rates of genome reorganization than most mammals. Moreover, as some of the ancestral syntenies may have been disrupted in the karyotype of *C*. *porcellus*, it is possible that they were also disrupted in the phylogenetic branch of Caviidae or, may be, in all hystricomorphs. To confirm or reject this hypothesis it is necessary to have a taxonomically rich array of hystricomorph species from different families.

### Future use of guinea pig probes in biomedical research

High-resolution synteny maps between human and laboratory mouse and rat, based on genome-wide sequence comparison, are available from ENSEMBL. The comparative chromosome map presented here should allow the inference of genome-wide chromosomal correspondence between guinea pig and laboratory mouse and rat, and many other species using human chromosome as the common reference, a great potential awaiting to be fully explored. Indeed, the breeding of the guinea pig as a pet is on the rise with over 20 breeds and many varieties involving coat color and hair properties being currently established, opening room to study pigmentation and hair growth related genes, their mutations and related disorders [71]. The economic importance of the guinea pig should not be ignored. They are an important meat source in South America that may represent interest for genetic characterization of meat-related genes.

## Conclusions

Here we present the first report on chromosome painting between human and hystricomorph rodents. Further comprehensive GTG-banding and chromosome painting studies between different representative species of this group would shed light on hystricomorph karyotype evolution and will help to establish phylogenetic relationships both within and between rodent suborders. The comparative chromosome map presented here is a starting point for further development of physical and genetic maps of the guinea pig and will facilitate the use of the guinea pig as a model for human diseases.

## Supporting Information

S1 Fig The G-banded ideogram complete with band nomenclature. p and q—short and long arms of chromosome, respectively. Bigger figures mark segments, smaller—separate bands.
(TIF)
