# Supplementary figures and images for: A First Generation Comparative Chromosome Map between Guinea Pig (Cavia porcellus) and Humans
Source: PLoS One. 2015 May 26;10(5):e0127937. doi: 10.1371/journal.pone.0127937 (PMC4444286; doi:10.1371/journal.pone.0127937)

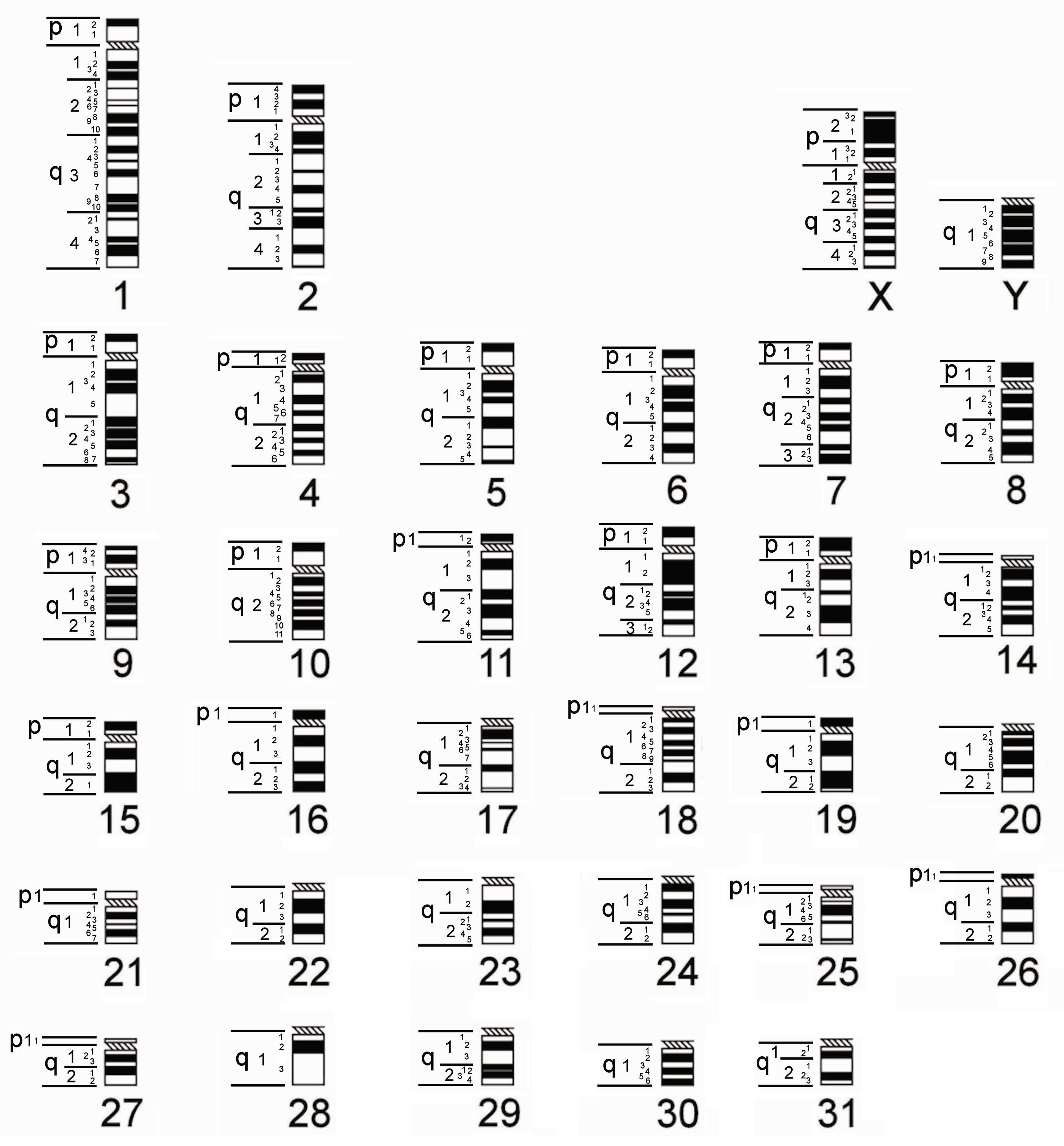

Supplement: S1 Fig — p and q—short and long arms of chromosome, respectively. Bigger figures mark segments, smaller—separate bands. (TIF) [file pone.0127937.s001.tif]
